# HIV-Associated Thrombotic Thrombocytopenic Purpura in a Virally Suppressed Patient With Normal CD4 Count: A Rare Presentation

**DOI:** 10.7759/cureus.69994

**Published:** 2024-09-23

**Authors:** Ammad Naeem, Ahsan Khan, Khawaja O Omar, Amir Kamran

**Affiliations:** 1 Hospital Medicine, Charleston Area Medical Center, Charleston, USA; 2 Hospital Medicine, WVU (West Virginia University) Medicine Thomas Memorial Hospital, Charleston, USA; 3 Internal Medicine, West Virginia School of Osteopathic Medicine, Lewisburg, USA; 4 Pulmonary and Critical Care Medicine, Charleston Area Medical Center, Charleston, USA; 5 Hematology and Oncology, Charleston Area Medical Center, Charleston, USA; 6 Hematology and Oncology, Allegheny Health Network, Pittsburgh, USA

**Keywords:** anemia, aquired ttp, autoimmune haemolytic anemia, cd4+ count, haart therapy, immune hematology, people living with hiv/aids, refractory ttp, thrombocytopenia, thrombotic thrombocytopenic thrombocytopenia

## Abstract

Thrombotic thrombocytopenic purpura (TTP) is a thrombotic process characterized by multiorgan failure secondary to microvascular thrombi comprising platelets and von Willebrand factor. HIV is a known risk factor for TTP. However, patients generally have low CD4+ count during initial presentation or subsequent flare-ups. This case report describes a 69-year-old man with HIV who presented with an initial presentation of TTP while having a prior history of HIV with a normal CD4 count and undetectable viral load on presentation. A 69-year-old Caucasian male with a previous history of HIV on antiretroviral therapy (ART), a history of recurrent deep vein thrombosis maintained on Coumadin, and no previous history of tobacco dependence or substance use presented to the emergency department with symptoms of fatigue and dyspnea worsening on exertion. The patient had stable vital signs on arrival. The initial lab workup was remarkable for hemoglobin of 9.3 g/dL, hematocrit 29%, platelets 22 x 10^9^/L, prothrombin time (PT) 51 seconds, activated partial thromboplastin time (PTT) 41 seconds, international normalized ratio (INR) 4.3 (on warfarin), blood urea nitrogen (BUN) 29 mg/dL, creatinine 2.0 mg/dL, total bilirubin 1.8 mg/dL, lactate dehydrogenase (LDH) 1229 U/L, haptoglobin less than 10 mg/dL, reticulocyte count 6.21%, fibrinogen 519 mg/dL, and D-dimer within normal limits. A clinical diagnosis of TTP was made with a peripheral blood smear showing schistocytes, as well as evidence of hemolytic anemia and thrombocytopenia. Prompt initiation of treatment with plasma exchange therapy was started. The patient was also given high-dose steroids and prednisone 1 mg/kg with a prolonged taper. Due to the delay in improvement in platelet count, the patient was also given concurrent rituximab therapy. TTP in HIV is rare, primarily seen in HIV patients who have a high viral load, low CD4 count, or if there is a delay in starting ART. In HIV-induced TTP patients, the relationship between CD4 count and viral load is complex and not fully elucidated. Further research is needed to understand better the interplay between HIV parameters (such as CD4 count and viral load) and the development of TTP in HIV-infected individuals who are already on treatment. This understanding could aid in identifying high-risk patients and developing targeted interventions to prevent or manage TTP in this population.

## Introduction

Thrombotic thrombocytopenic purpura (TTP) is a rare but life-threatening disorder characterized by microangiopathic hemolytic anemia, thrombocytopenia, and ischemic end-organ damage [1]. Despite its low prevalence, TTP poses significant diagnostic and therapeutic challenges due to its diverse etiologies and variable clinical presentations [2]. 

In TTP, microthromboses result from the buildup of von Willebrand factor (VWF) due to ADAMTS-13 deficiency. This leads to the formation of obstructive ultra-large VWF multimers, causing hemolytic anemia and thrombocytopenia. ADAMTS-13 deficiency can be congenital or acquired, associated with conditions like malignancies, autoimmune diseases, and infections such as HIV [1]. The true pathophysiology of ADAMTS-13 deficiency in HIV patients is not clearly understood but is believed to be multifactorial. Some studies suggest that chronic inflammation and complement activation likely contribute to the development of HIV-associated TTP through endothelial dysfunction [3,4]. In addition to ADAMTS-13 deficiency and endothelial dysfunction, secondary triggers such as opportunistic infections (e.g., cytomegalovirus, *Mycobacterium tuberculosis*) and inflammatory conditions (e.g., immune reconstitution inflammatory syndrome (IRIS)) can further exacerbate the prothrombotic state in HIV patients predisposed to TTP [3].

The primary treatment for TTP is plasma exchange therapy (PLEX), which removes autoantibodies and replenishes deficient ADAMTS-13 enzyme. Additionally, corticosteroids are often used to suppress immune-mediated destruction of ADAMTS-13 in refractory cases [5]. Rituximab, a monoclonal antibody targeting B cells, is employed for refractory or relapsed cases although not approved for TTP yet [5,6]. In severe cases or those with neurologic involvement, immunosuppressive agents like cyclophosphamide can be used as immunosuppressants [5,7]. Caplacizumab (Cablivi), a nanobody targeting vWF, was FDA-approved in 2019 for treating acquired thrombotic thrombocytopenic purpura (aTTP) alongside plasma exchange and immunosuppressive therapy. It reduces time to platelet count response and lowers aTTP-related mortality, recurrence, and major thromboembolic events [8]. Prompt initiation of treatment is crucial to prevent end-organ damage and reduce mortality. Treatment in HIV-induced TTP remains the same, prompt initiation of antiretroviral therapy (ART) is key in ART-naïve patients, and optimization of ART is important in virally suppressed HIV patients to prevent relapses [3].

HIV is a known risk factor for TTP; however, patients generally have low CD4+ count during initial presentation or subsequent flare-ups [1]. HIV can increase the risk of developing TTP by 15-40 times. Other risk factors for TTP in HIV patients include higher HIV-1 RNA levels, clinical AIDS, opportunistic infections, drugs, and immune reconstitution after highly active antiretroviral therapy (HAART). This case report describes a 69-year-old man with diagnosed HIV maintained on HAART, who presented with an initial presentation of TTP while having a normal CD4 count and undetectable viral load (HIV-1 RNA levels) on presentation.

## Case presentation

A 69-year-old Caucasian male with a known history of HIV on ART and recurrent deep vein thrombosis managed with Coumadin presented to the emergency department due to worsening fatigue and dyspnea on exertion. He had no history of tobacco or substance use. On arrival, his vital signs were stable, and initial imaging, including a chest X-ray, showed no acute abnormalities. However, extensive lab work revealed concerning results: hemoglobin 9.3 g/dL, hematocrit 29%, platelets 22 x 10^9^/L, prothrombin time (PT) 51 seconds, partial thromboplastin time (PTT) 41 seconds, international normalized ratio (INR) 4.3 (on warfarin), blood urea nitrogen (BUN) 29 mg/dL, creatinine 2.0 mg/dL, total bilirubin 1.8 mg/dL, lactate dehydrogenase (LDH) 1229 U/L, haptoglobin less than 10 mg/dL, reticulocyte count 6.21%, fibrinogen 519 mg/dL, with D-dimer within normal limits. Lab work pertinent to the case and diagnosis revealed concerning results, which are summarized in Table 1.

**Table 1:** Laboratory Parameters Over Time With Reference Ranges and Units

Parameter	Unit	Reference Range	Day 1	Day 2	Day 3	Day 4	Day 5	Day 6
Hemoglobin	g/dL	13.8-17.2	9.3	8.7	8.9	8.1	7.9	8.0
Hematocrit	%	40.7-50.3	29.5	28.0	27.8	26.4	25.7	26.8
Platelets	x10^3^/µL	150-450	22	19	61	133	164	199
Creatinine	mg/dL	0.7-1.3	2.01	1.84	1.44	1.56	1.44	1.44
ADAMTS-13	%	50-150	N/A	<2	N/A	N/A	N/A	7.36

**Figure 1:**
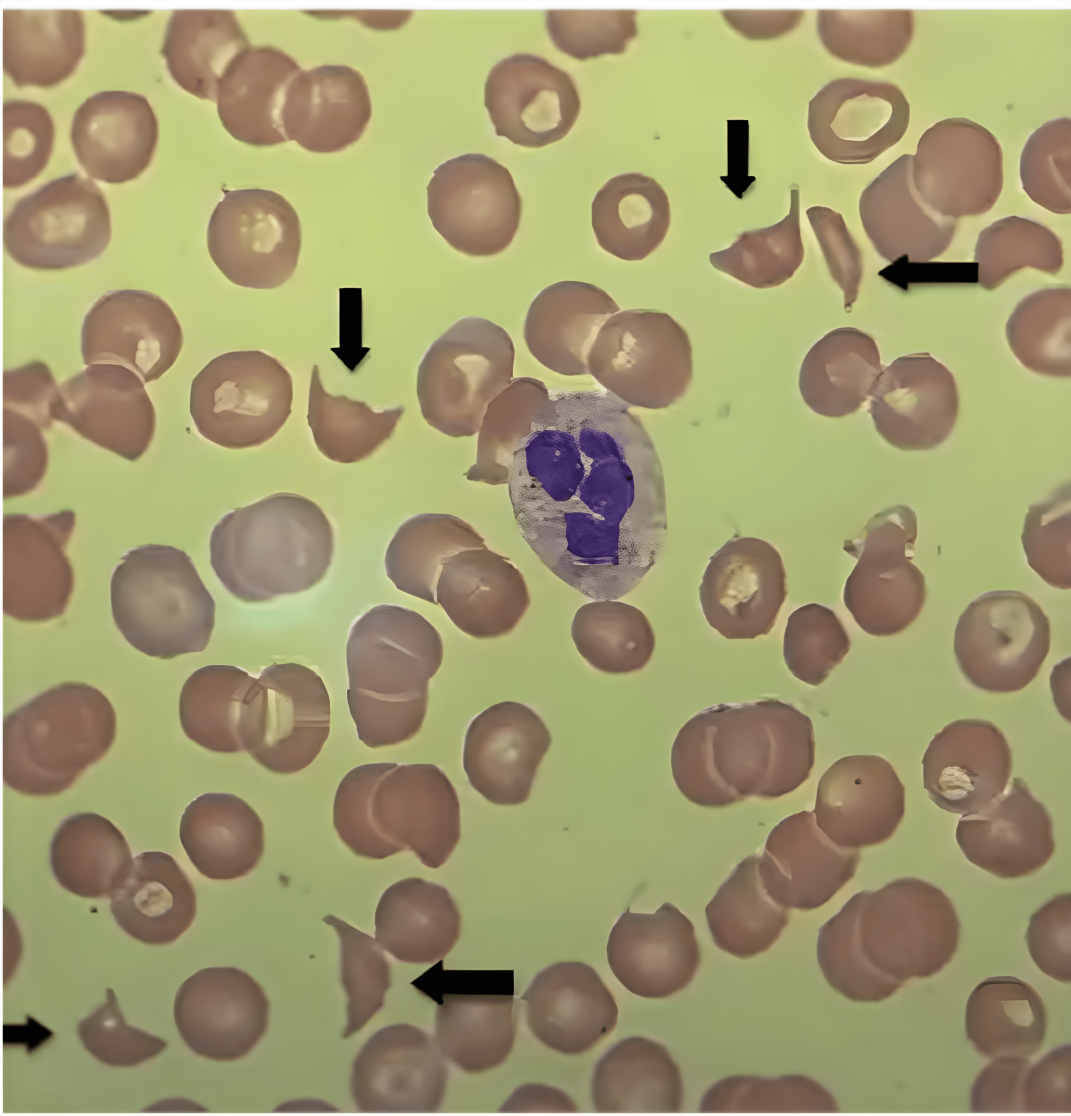
Peripheral Blood Smear Showing Schistocytes (arrows)

Based on peripheral blood smear findings of schistocytes (as seen in Figure 1), along with hemolytic anemia and thrombocytopenia, a clinical diagnosis of TTP was established. Despite presenting with thrombocytopenia, microangiopathic hemolytic anemia, and renal dysfunction, the patient did not exhibit fever or neurological abnormalities. Immediate treatment with PLEX was initiated, alongside high-dose steroids (prednisone 1 mg/kg daily with a prolonged taper) to mitigate the immune response and reduce inflammation. Due to the delayed response in platelet count improvement, rituximab therapy was administered concurrently, with the patient receiving one dose during hospitalization and a plan set for continued infusions over four weeks.

Pre-treatment ADAMTS-13 levels were confirmed to be less than 2% (normal range: 50-150%), consistent with the diagnosis of TTP. Post-treatment reassessment showed normalization of ADAMTS-13 levels to 96%, indicating a favorable response to therapy. Throughout this period, the patient's ART was briefly paused to facilitate diagnosis and promptly reinstated upon stabilization. Notably, his CD4 count remained above 400 cells/mm³ (normal range: 500-1600 cells/mm³), with an undetectable viral load, underscoring the effectiveness of ongoing HIV management despite the acute medical challenge posed by TTP. The case highlights the complexity of managing concurrent medical conditions in HIV-positive patients and the critical role of prompt diagnosis and multidisciplinary treatment in improving clinical outcomes.

## Discussion

TTP has many etiologies, including infectious etiologies, which can be enteric pathogens, viruses, or fungi. Other etiologies include autoimmune conditions, medications, or miscellaneous conditions such as pregnancy. Delayed diagnosis and treatment can lead to mortality, which can be as high as 90% [9]. Commonly, microangiopathic hemolytic anemia with thrombocytopenia is seen without organ damage. Thrombocytopenia is commonly seen in HIV patients; according to the literature, about 6-15% of HIV-positive patients develop thrombocytopenia, which can be asymptomatic; multiple mechanisms cause it, and TTP is one of them [10]. TTP in HIV is rare, primarily seen in HIV patients who have a high viral load, low CD4 count, or if there is a delay in starting ART. In HIV-induced TTP patients, the relationship between CD4 count and viral load is complex and not fully elucidated. Generally, HIV infection leads to a decrease in CD4 count and an increase in viral load. However, the impact of these HIV-related parameters on the development or progression of TTP may vary among individuals. Some studies suggest that lower CD4 counts are associated with an increased risk of TTP in HIV-infected individuals.

Additionally, higher viral loads may contribute to immune dysregulation and endothelial damage, potentially exacerbating the risk of TTP. IRIS is a condition that can occur in individuals with HIV shortly after starting ART. While IRIS-related thrombocytopenia is uncommon, clinicians should consider it in the differential diagnosis. In this case, the patient was already on ART and had an undetectable viral load, making IRIS-related thrombocytopenia less likely, favoring TTP diagnosis in rare instances of normal CD4 count. There have been rare instances of TTP in an HIV individual on ART who is virally suppressed with a normal CD4 count. However, the exact mechanisms underlying this relationship remain unclear and may involve various factors, including immune system dysfunction, viral replication, and inflammatory processes. According to a five-year study conducted at a tertiary care center in sub-Saharan Africa, TTP was the initial presenting symptom of HIV infection in a considerable number of cases, with some patients being noncompliant with ART. The majority of study participants were Black and predominantly female [11]. Hence, it opens the discussion window of pathophysiology other than high viral load-induced endothelial damage, low CD4 count-induced immune activation, or secondary triggers such as opportunistic infections. Hence, thrombocytopenia seen in an HIV patient should be taken seriously, as there are many etiologies, and some patients can be asymptomatic. However, a missed diagnosis of TTP can lead to a delay in treatment, which can lead to a high mortality rate. Prompt diagnosis is essential, so patients can be referred to a higher center if treatments are unavailable. A multidisciplinary team and advanced therapeutic resources can greatly enhance patient outcomes for this life-threatening illness.

## Conclusions

Further research is needed to better understand the interplay between HIV parameters (such as CD4 count and viral load) and the development of TTP in HIV-infected individuals who are already on treatment. This understanding could aid in the identification of high-risk patients and the development of targeted interventions to prevent or manage TTP in this population.
